# Supplementary material for: Droplet-based high-throughput cultivation for accurate screening of antibiotic resistant gut microbes
Source: eLife. 2020 Jun 17;9:e56998. doi: 10.7554/eLife.56998 (PMC7351490; doi:10.7554/eLife.56998)
Supplement: Transparent reporting form [file elife-56998-transrepform.docx]

***eLife’s* transparent reporting form**

We encourage authors to provide detailed information *within their submission* to facilitate the interpretation and replication of experiments. Authors can upload supporting documentation to indicate the use of appropriate reporting guidelines for health-related research (see [EQUATOR Network](http://www.equator-network.org/%20)), life science research (see the [BioSharing Information Resource](https://biosharing.org/)), or the [ARRIVE guidelines](http://www.plosbiology.org/article/info:doi/10.1371/journal.pbio.1000412) for reporting work involving animal research. Where applicable, authors should refer to any relevant reporting standards documents in this form.

If you have any questions, please consult our Journal Policies and/or contact us: [editorial@elifesciences.org](mailto:editorial@elifesciences.org).

**Sample-size estimation**

- You should state whether an appropriate sample size was computed when the study was being designed
- You should state the statistical method of sample size computation and any required assumptions
- If no explicit power analysis was used, you should describe how you decided what sample (replicate) size (number) to use

Please outline where this information can be found within the submission (e.g., sections or figure legends), or explain why this information doesn’t apply to your submission:

All samples within this submission are summarized on the Experiment_Info tab of the Supp_Info_and_Data excel sheet. We analyzed our data for two separate experiments – Media testing (Figs. 2-5) and Antibiotic testing (Fig. 6). For each of these two separate experiments, we compare droplet and plate cultures. All statistical analysis is described in “Data Analysis” in Methods.

We observed no significant variation in the community composition between biological replicates or cultivation time for either droplets or plates (Supp. Fig. 5). Therefore, the number of samples we chose was at least 3 averaged over just media or antibiotics for droplets or plates (i.e., excluding cultivation time).

**Replicates**

- You should report how often each experiment was performed
- You should include a definition of biological versus technical replication
- The data obtained should be provided and sufficient information should be provided to indicate the number of independent biological and/or technical replicates
- If you encountered any outliers, you should describe how these were handled
- Criteria for exclusion/inclusion of data should be clearly stated
- High-throughput sequence data should be uploaded before submission, with a private link for reviewers provided (these are available from both GEO and ArrayExpress)

Please outline where this information can be found within the submission (e.g., sections or figure legends), or explain why this information doesn’t apply to your submission:

Cultures in this experiment were started over 7 different days, from multiple different stool aliquots (of the same sample). All experiments were biologically independent. For plate experiments, this means we spread aliquoted sample onto separate plates. For droplets, biologically independent means the aliquot was diluted, loaded into a syringe, and droplets were formed for each experiment. No technical replicates were performed in this experiment. (Which we define as collecting DNA multiple times from one biologically independent experiment. I.e., for a 2x technical replicate on plates, this would mean scraping DNA into 2 separate vials, and doing 2 DNA extractions, and 2 16S sequencing runs). However, we filter our amplicon sequence variants using a conservative filtering strategy so that technical variation and noise are minimized.

Prototype experiments for droplet generation and culture (which were not sequenced) are not included in this data. All other data was included which was successfully cultured and sequenced.

**Statistical reporting**

- Statistical analysis methods should be described and justified
- Raw data should be presented in figures whenever informative to do so (typically when N per group is less than 10)
- For each experiment, you should identify the statistical tests used, exact values of N, definitions of center, methods of multiple test correction, and dispersion and precision measures (e.g., mean, median, SD, SEM, confidence intervals; and, for the major substantive results, a measure of effect size (e.g., Pearson's r, Cohen's d)
- Report exact p-values wherever possible alongside the summary statistics and 95% confidence intervals. These should be reported for all key questions and not only when the p-value is less than 0.05.

Please outline where this information can be found within the submission (e.g., sections or figure legends), or explain why this information doesn’t apply to your submission:

All statistical analysis is described in “Data Analysis” in Methods.

#1. Mann-Whitney U test (Fig. 2b-c, 3b, & Supp. Fig. 8 a-c).

#2. Kolmogorov-Smirnov test (Fig. 5a & Supp. Fig 3).

#3. Hierarchical cluster analysis with multiscale bootstrap resampling (Fig. 4)

(For large datasets, or papers with a very large number of statistical tests, you may upload a single table file with tests, Ns, etc., with reference to sections in the manuscript.)

**Group allocation**

- Indicate how samples were allocated into experimental groups (in the case of clinical studies, please specify allocation to treatment method); if randomization was used, please also state if restricted randomization was applied
- Indicate if masking was used during group allocation, data collection and/or data analysis

Please outline where this information can be found within the submission (e.g., sections or figure legends), or explain why this information doesn’t apply to your submission:

We grouped samples into droplets and plates, as this formed the central question of our research – Do droplets improve the cultivation of organisms from human stool and how can we apply this to better understand gut microbial communities? We tested this grouping across different media, cultivation time, and antibiotic usage.

**Additional data files (“source data”)**

- We encourage you to upload relevant additional data files, such as numerical data that are represented as a graph in a figure, or as a summary table
- Where provided, these should be in the most useful format, and they can be uploaded as “Source data” files linked to a main figure or table
- Include model definition files including the full list of parameters used
- Include code used for data analysis (e.g., R, MatLab)
- Avoid stating that data files are “available upon request”

Please indicate the figures or tables for which source data files have been provided:

All data plotted is included in the Excel Supp_Info_and_Data file. This includes the raw counts for each ASV, the filtered ASVs (in percentages), the sequences, the assigned taxonomy, ecological measures (richness, diversity, etc.), oligotype sequences, and oligotype taxonomy. We also include the analysis code for filtering ASVs (Script: ASV_Filtering.m // Data: ASV_Counts_Matrix table (subtab of the excel file)). The oligotyping and Minimum Entropy Decomposition code used for analyzing raw Illumina 16S reads is available at <http://merenlab.org/>
